# SARS-CoV-2 Persistence in Immunocompromised Patients Requiring Treatment With Convalescent Plasma: A Case Report

**DOI:** 10.7759/cureus.54564

**Published:** 2024-02-20

**Authors:** Gabrielle Librizzi, Viraj Modi, Audun J Lier

**Affiliations:** 1 Internal Medicine, Stony Brook University Hospital, Stony Brook, USA; 2 Internal Medicine, Northport Veterans Affairs Medical Center, Northport, USA; 3 Infectious Diseases, Northport Veterans Affairs Medical Center, Northport, USA; 4 Infectious Diseases, Stony Brook University Hospital, Stony Brook, USA

**Keywords:** covid-19, follicular lymphoma, cycle threshold, convalescent plasma, immunosuppressed

## Abstract

Severe acute respiratory syndrome-2 (SARS-CoV-2) infection in immunocompromised patients presents a challenge, as patients with such conditions may have severe courses. Identifying modalities to shorten the course or lessen the severity of infection could be potentially beneficial.

A 76-year-old male with follicular lymphoma on rituximab and lenalidomide presented with COVID-19 pneumonia requiring intensive care unit (ICU) level care for persistent hypoxemia. He was treated with an extended course of remdesivir, as recommended by the Infectious Diseases service, but he maintained a persistently high viral load, necessitating a delay of his cancer treatment until he had recovered from his infection. On hospital day 31, he was given one dose of convalescent plasma with improvement in his SARS-CoV-2 viral load. He was able to be discharged and resumed cancer treatment soon thereafter.

Convalescent plasma is a potential therapeutic option for immunocompromised patients with SARS-CoV-2 infection and should be considered early in the hospital course. Additionally, cycle threshold monitoring may be beneficial in certain scenarios: for instance to guide consideration of alternative therapies in patients with severe COVID-19 who have persistent symptoms and viremia while on guideline-directed therapy.

## Introduction

Severe acute respiratory syndrome-2 (SARS-CoV-2) is a respiratory virus that can lead to severe morbidity and mortality in persons who are immunocompromised [1]. Further, prolonged infectious courses have been documented in persons who are immunocompromised, which may present a management challenge for persons who often require resolution of their infection prior to reinitiating chemotherapy or other immunomodulating therapy [2].

## Case presentation

Here we present the case of a 76-year-old male with a past medical history significant for follicular lymphoma, essential thrombocytosis, hypertension, and hyperlipidemia who presented to the Northport Veterans Affairs Medical Center, USA with a complaint of fatigue, myalgias, nasal congestion and night sweats. The patient endorsed exposure to SARS-CoV-2 via his son who had recently tested positive with a home antigen test and two days prior to presentation, he tested positive for SARS-CoV2 via a home antigen testing kit. He had previously received the primary (two-dose) Pfizer mRNA vaccine series as well as one Pfizer mRNA booster one year prior. Three years prior to presentation, he was diagnosed with follicular lymphoma. He had initially achieved remission, discovered via a computerized axial tomography (CT) scan, with a combination therapy of bendamustine and rituximab. However, one year later he underwent a repeat positron emission tomography (PET) scan which showed an enlarged right groin lymph node, later identified as a CD10-positive B-cell lymphoma on biopsy. Subsequently, he began a clinical trial, but a repeat CT scan showed disease progression three months prior to presentation, thus he was initiated on a different treatment regimen: rituximab and lenalidomide. He last received a dose of rituximab one week prior to presentation.

In the emergency department (ED), he had vitals as shown in Table 1 and laboratory studies as shown in Table 2.

**Table 1:** Emergency Department Vitals mmHg = millimeters of mercury; bpm= beats per minute/breaths per minute

Vital Sign	
Temperature (degrees C)	37.2
Blood Pressure (mmHg)	145/81
Heart Rate (bpm)	89
Respiratory Rate (bpm)	18
Pulse Oximetry (%)	87

**Table 2:** Emergency Department Pertinent Labs

Lab Value	
White Blood Cell Count (per mm^3^)	7.9
Red Blood Cell Count (per mm^3^)	5.38
Platelet Count (per mm^3^)	419
Lactic Acid	2.3

He tested positive for SARS-CoV-2 on a real-time polymerase chain reaction (PCR) test obtained from a nasal swab specimen (Xpert® Xpress CoV-2, Cepheid platform, Cepheid, Sunnyvale, USA). A chest X-ray revealed bilateral coarsening of the bronchovascular lung markings, but no consolidation, pleural effusion, or pulmonary vascular congestion. He was placed on three liters of supplemental oxygen with improvement in oxygen saturation to 93%, was initiated on intravenous (IV) remdesivir and oral dexamethasone therapy and received one litre of IV normal saline, and an albuterol-ipratropium combination nebulizer with admission to the inpatient general medicine service.

His hospital course was complicated by worsening acute hypoxemic respiratory failure requiring high-flow supplemental oxygen with an upgrade to the intensive care unit (ICU) for closer monitoring. He was also initiated on empiric cefepime and vancomycin on hospital day 4 while undergoing a workup for concomitant hospital-acquired pneumonia. On hospital day 9, due to persistent hypoxemia, the Infectious Diseases (ID) service was consulted to assist with management. On review of his SARS-CoV-2 PCR testing obtained on day 9, he was found to have a cycle threshold of 24 (Xpert® Xpress CoV-2, Cepheid platform). Due to his persistent hypoxemia and low cycle threshold, an extension of IV remdesivir was pursued, supported by a previous case report describing successful treatment of SARS-CoV-2 infection following a 30-day course of IV remdesivir in a patient with secondary hypogammaglobulinemia who had persistently low PCR cycle thresholds and remained symptomatic [3]. Further, it was recommended by the ID service not to pursue either baricitinib or tocilizumab therapy as both his erythrocyte sedimentation rate (ESR) and C-reactive protein (CRP) were low. Due to low suspicion of bacterial infection (negative blood and sputum cultures), both vancomycin and cefepime were discontinued on hospital day 10. He remained in the ICU for two weeks requiring a high flow nasal cannula and daily IV remdesivir therapy with cycle threshold values ranging between 20 and 30 (Cepheid platform), indicating that he was still likely infectious and thus his cancer treatment remained on hold. On hospital day 27, he was able to be weaned to a nasal cannula and downgraded to the inpatient general medical floors. However, cycle thresholds repeated every two to three days were persistently less than 30 (Cepheid platform) despite IV remdesivir therapy (see Table 3 for a range of values).

**Table 3:** Trend in Cycle Threshold Values Over the Course of the Hospital Course * Value before MICU (Medical Intensive Care Unit) upgrade ** Value before downgraded to general medicine floors *** Value before given convalescent plasma

Hospital Day	Cycle Threshold (CT)
9	24
11	20.4
14	29.7
16	27
20^*^	27
23	23
26^**^	24
29	26.7
31^***^	33.6
32	40.4

Over the course of this admission, while his cancer treatment had been held by the outpatient oncology team due to his persistent viremia, the patient expressed to the inpatient medical team that he desired to restart his cancer treatment as soon as possible. Due to the urgent nature of restarting chemotherapy and the need to resolve his infection beforehand, a decision was made to administer a dose of convalescent plasma. On hospital day 31, a dose of convalescent plasma was administered and a repeat cycle threshold (Cepheid platform), obtained on the following day, noted an increase to 40.4. The patient also noted the resolution of his COVID-19 symptoms, which included shortness of breath and cough. The patient was subsequently discharged home two days after receipt of plasma infusion. He was able to restart cancer treatment (lenalidomide and rituximab) 11 days after hospital discharge. The SARS-CoV-2 strain was sequenced and found to be 22E Omicron clade, BQ.1 lineage.

The patient followed up at the Northport VA post-COVID outpatient clinic and, during visits at one month and five months post-discharge, continued to note episodes of intermittent dyspnea on exertion as well as brain fog. However, at one-year follow-up, he noted a complete resolution of his post-COVID symptoms.

## Discussion

This case demonstrates the inherent therapeutic management challenge that severe COVID-19 may present in immunocompromised persons. First, this case highlights the potential limitation of remdesivir and dexamethasone in immunocompromised patients, but also highlights a promising alternative treatment avenue for this vulnerable population: convalescent plasma.

Although our patient received dexamethasone and an extended course (30 days) of IV remdesivir, his SARS-CoV-2 cycle threshold was persistently low until he received convalescent plasma. One potential explanation for this phenomenon is the development of de novo remdesivir resistance. A previous case report published in 2022 highlighted a de-novo remdesivir resistance mutation which occurred during treatment of persistent SARS-CoV-2 infection in a patient with an acquired B-cell deficiency [4]. This patient had received IV remdesivir for a protracted course of severe COVID-19. Ten days (extended from five days) of remdesivir transiently produced a virologic response, but the patient experienced a recurrence of viral shedding. At this time, whole genome sequencing was performed and identified a mutation (E802D) that was not present in a pre-treatment specimen; in vitro testing of this specimen demonstrated a six-fold increase in remdesivir IC50. This patient was eventually successfully treated with casivirimab-imdevimab, illustrating the potential for remdesivir resistance to have a clinical effect. In our patients’ case, persistent viremia in the setting of an impaired immune system may have introduced selective pressure to generate a de novo mutation that conferred increased resistance to remdesivir. Another case report published in January 2024 described a patient with persistent SARS-CoV-2 infection with viral evolution during their prolonged course. In this patient, there were 14 mutations identified, three of which (S494P, T22I, and T95A) have been associated with antibody neutralization [5]. Unfortunately, despite requests to multiple laboratories, we were unable to do any genotypic testing for de novo remdesivir mutations. Our case also highlights the beneficial use of cycle threshold monitoring in specific clinical scenarios, for instance, to guide consideration of alternative therapies in patients with severe COVID-19 who have persistent symptoms while on guideline-directed therapy. Cycle thresholds have been used to guide isolation status, as cycle threshold and infectivity are inversely proportional: the lower the cycle threshold the more infectious an individual may be considered to be. While there is no hard cutoff for a person to be deemed “non-infectious”, a value of 40 is often used as the cutoff to deem a test result positive: anything below 40 is positive [6]. Further, it is vital to note that the same Cepheid platform was used throughout the patient’s hospital course, thus a consistent interpretation of our patients’ SARS-CoV-2 viral load could be made. In our case, repeat cycle thresholds demonstrated that remdesivir monotherapy was not effective in eradicating his SARS-CoV-2 infection, and did support the efficacy of convalescent plasma, as evidenced by a significant rise in the SARS-CoV-2 cycle threshold after one dose of convalescent therapy. While useful in certain clinical scenarios, cycle thresholds should not be used to guide management for all patients with COVID-19. Moreover, a meta-analysis published in January 2023 demonstrated a mortality benefit when using convalescent plasma in immunocompromised patients (risk ratio [RR], 0.63 [95% CI, 0.50-0.79]) [7]. Therefore, not only could convalescent plasma be useful in clearing SARS-CoV-2, but it could also decrease mortality in this patient population.

## Conclusions

In summary, our case report adds to the literature that dexamethasone and remdesivir alone might not be able to overcome SARS-CoV-2 viremia in immunosuppressed patients. It is imperative to find ways to shorten the course of SARS-CoV-2 infection in patients like ours so they can continue cancer treatment as soon as possible and to reduce the selective pressure of SARS-CoV-2 to overcome remdesivir, a highly effective antiviral therapy that has been the cornerstone of treatment of severe COVID-19 and prevention of COVID-19 in high-risk populations. In these cases, the use of SARS-CoV-2 PCR cycle thresholds may guide our decision-making and in particularly in protracted courses, the use of convalescent plasma may be considered. Our patient received convalescent plasma greater than one month after remdesivir initiation, which then caused his cycle threshold to increase above 40. The up-trending cycle threshold values correlated with improved symptoms, including being weaned off of supplemental oxygen. Going forward, convalescent plasma in conjunction with remdesivir and dexamethasone may be considered early in the hospital course in immunocompromised patients to reduce viremia and may also potentially decrease the selective pressure on the virus to mutate.

## References

[1] Fung M, Babik JM (2023). COVID-19 in immunocompromised hosts: what we know so far. Clin Infect Dis.

[2] Magyari F, Pinczés LI, Páyer E (2022). Early administration of remdesivir plus convalescent plasma therapy is effective to treat COVID-19 pneumonia in B-cell depleted patients with hematological malignancies. Ann Hematol.

[3] Martinez MA, Chen TY, Choi H (2022). Extended Remdesivir infusion for persistent coronavirus disease 2019 infection. Open Forum Infect Dis.

[4] Gandhi S, Klein J, Robertson AJ (2022). De novo emergence of a remdesivir resistance mutation during treatment of persistent SARS-CoV-2 infection in an immunocompromised patient: a case report. Nat Commun.

[5] Overbeck V, Taylor BP, Turcinovic J (2024). Successful treatment of SARS-CoV-2 in an immunocompromised patient with persistent infection for 245 days: a case report. Heliyon.

[6] Tom MR, Mina MJ (2020). To interpret the SARS-COV-2 test, consider the cycle threshold value. Clin Infect Dis.

[7] Senefeld JW, Franchini M, Mengoli C (2023). COVID-19 convalescent plasma for the treatment of immunocompromised patients: a systematic review and meta-analysis. JAMA Netw Open.

